# Reinvigorating the Cancer-Immunity Cycle by Intratumoral Administration of Conventional Dendritic Cells in Melanoma and Other Solid Tumors: A Narrative Review

**DOI:** 10.3390/vaccines14050402

**Published:** 2026-04-30

**Authors:** Manon Vounckx, Iris Dirven, Cleo Bertels, Julia Katharina Schwarze, Xenia Geeraerts, Sandra Tuyaerts, Anaïs Boisson, Karen Willard-Gallo, Bart Neyns

**Affiliations:** 1 Laboratory of Medical and Molecular Oncology (LMMO), Department of Medical Oncology, UZ Brussel, Vrije Universiteit Brussel (VUB), 1090 Brussels, Belgiumxenia.geeraerts@vub.be (X.G.); sandra.tuyaerts@vub.be (S.T.); bart.neyns@uzbrussel.be (B.N.); 2 Medical and Molecular Oncology, Translational Oncology Research Center (TORC), Brussels Health Campus, Faculty of Medicine and Pharmacy, Vrije Universiteit Brussel (VUB), 1090 Brussels, Belgium; 3 Molecular Immunology Unit (MIU), Institut Jules Bordet, Université Libre de Bruxelles, 1070 Brussels, Belgium

**Keywords:** dendritic cell therapy, intratumoral therapy, combinatorial cancer immunotherapy, immune checkpoint inhibition

## Abstract

Dendritic cells (DCs) are central to cancer immunity, orchestrating both innate and adaptive immune responses. In melanoma and other solid tumors, however, their function is often impaired within the tumor microenvironment (TME), leading to weakened antitumor immunity and diminished responses to immune checkpoint inhibitors (ICIs) and adoptive tumor-infiltrating lymphocyte (TIL) therapy. Among the various cell-based immunotherapy approaches, DC therapy—particularly using blood-derived conventional DCs (cDCs)—holds considerable promise. Compared with traditional monocyte-derived DCs (moDCs), cDCs exhibit superior antigen processing and cross-presentation capacities. The therapeutic application of cDCs was initially pioneered in vaccine strategies involving ex vivo antigen loading and maturation, followed by administration to lymph nodes. More recently, intratumoral (IT) cDC immunotherapy has emerged as a strategy to reinvigorate the cancer-immunity cycle by engaging the full repertoire of tumor-associated antigens while limiting systemic toxicity. This review discusses the underlying biological mechanisms and summarizes the clinical outcomes of IT DC therapy in cancer. Notably, combination approaches incorporating IT cDCs with ICIs, oncolytic viruses, synthetic adjuvants, radiation, or cryotherapy are emerging as promising strategies to overcome both primary and acquired resistance to ICI monotherapy. Collectively, these findings highlight the potential of integrating IT cDC therapy with complementary immunotherapies in next-generation, cross-tumor treatment strategies.

## 1. Introduction

Over the past decade, systemic immune checkpoint inhibition (ICI, incl. PD-(L)1, CTLA-4 and LAG-3 receptors) by intravenous administration of medicinal monoclonal antibodies (mAb) has transformed at first the treatment paradigm for metastatic melanoma and later on also for many other solid and hematological cancers [1,2,3,4,5,6,7]. This revolution highlighted the potential of the immune system to serve as a weapon in the battle against cancer. However, most cancer patients either do not respond to ICIs upfront due to primary resistance or eventually develop acquired resistance leading to tumor progression [8]. A central factor driving the variability in outcomes to ICI is the tumor’s “foreignness” and “immunogenicity” reflected by the tumor mutation burden (TMB), hence presence of highly immunogenic neoantigens in particular, and the baseline “cancer immune set point”—specifically, whether the tumor is T-cell inflamed (“hot”), or not (“cold”) [9]. “Hot tumors”, more prevalent among malignancies such as melanoma and non-small cell lung carcinoma (NSCLC), often spontaneously elicit a potent adaptive immune response, reflected by an infiltration of cytotoxic CD8^+^ T-cells, and consequently respond well when unleashed by ICI.

However, even initially responsive tumors may acquire resistance by engaging alternative immune evasion mechanisms, causing a major clinical challenge [10,11]. These include sustained upregulation of PD-L1 and other immune suppressive checkpoint molecules (e.g., LAG-3, T-cell immunoglobulin and mucin-domain containing-3 [TIM-3], and T-cell immunoreceptor with Ig and ITIM domains [TIGIT]) leading to T-cell exhaustion and dysfunction, and alterations in the antigen presentation machinery (e.g., mutations in β2-microglobulin), or disruption of the JAK/STAT and IFNγ signaling cascades, impairing recognition and elimination by activated cytotoxic T-cells. “Cold tumors” are essentially characterized by absent or minimal immune infiltration within tumor nests (classified as T-cell-excluded phenotypes, when T-cells are restricted to the tumor periphery; or desert phenotypes, when T-cells are completely absent) and are typically unresponsive to ICI, reflecting primary resistance. These tumors are typically devoid of conventional dendritic cells (cDCs) and are most often associated with low TMB and downregulated major histocompatibility complex (MHC) class I expression. They exist within a hostile tumor microenvironment (TME) characterized by high levels of immunosuppressive cytokines and frequent enrichment of M2-like-polarized macrophages and other cancer-associated suppressive cells, collectively reinforcing immune evasion and resistance to therapy [12,13]. Understanding and reshaping this immune contexture is therefore key to overcoming resistance and improving the efficacy of cancer immunotherapy.

The central role of professional antigen-presenting cells (APC), especially dendritic cells (DCs), in cancer immunity has been recognized for many years and remains a subject of research efforts. Initially, from a therapeutic perspective, so-called monocyte-derived dendritic cells (moDCs) were considered optimal formulations for immunizing patients against tumor-associated antigens (TAA) (so-called “nature’s adjuvant”). Such formulations were generated ex vivo from peripheral blood mononuclear cells using GM-CSF and IL-4, and have been widely used in clinical trials [14]. After antigen loading, typically with peptides, RNA, or tumor lysates, these cells are administered by injection to the skin, lymph nodes, or intravenously to elicit antigen-specific T-cell responses. moDC vaccines have consistently demonstrated safety, feasibility, and immunogenicity, yet their clinical efficacy remains modest, largely due to limited migratory capacity and functional exhaustion [15,16,17,18,19]. While generally ineffective as monotherapy, prior moDC vaccination has been linked to enhanced responses to PD-1 blockade [15]. To date, only sipuleucel-T (Provenge^®^)—an autologous ex vivo DC vaccine— has achieved significant efficacy in phase III trials for metastatic castration-resistant prostate cancer and was approved by the FDA and EMA for this indication [20]. Combinatorial strategies using moDCs have produced durable remissions, even in patients refractory to ICI [21,22]. However, limitations persist—some trials show no additional benefit when moDCs are combined with chemotherapy (e.g., cisplatin), and immune escape may be linked to dysregulated Inducible T-cell Co-Stimulator Ligand (ICOSL) signaling on DCs [23]. Overall, their impact in the setting of melanoma remains constrained, largely reflecting impaired migration to lymphoid tissues and functional exhaustion that limits T-cell priming [24].

More recently, an essential physiological role has been attributed to cDCs in orchestrating the cancer immune response, putting them at the focus of innovative combinatorial treatment strategies, with clinical evidence largely derived from melanoma and only limited, emerging data available for other solid tumor types, as addressed in this review.

## 2. Conventional Dendritic Cells: Essential Orchestrators of Anti-Cancer-Immunity

DCs serve as the gatekeepers of cancer immunity, playing a central role in initiating and sustaining antitumor immune responses through “licensing” T-cell activity within the TME. Recruited to the TME by innate immune cues, DCs function as professional APCs by capturing and processing tumor antigens, which they subsequently present to naïve T-cells in tumor-draining lymph nodes. Through the formation of “triadic” interactions with CD4^+^ and CD8^+^ T-cells, DCs provide the signals necessary to prime both “license” effective antitumor T-cell expansion and activity [25,26,27]. Yet, within the TME, tumors exploit multiple mechanisms—such as secretion of TGF-β, IL-10, and VEGF, and recruitment of regulatory T-cells (Tregs) and myeloid-derived suppressor cells (MDSCs)—to impair DC migration, differentiation, and activation [28]. Moreover, tumor-intrinsic activation of the WNT–β-catenin pathway can promote DC exclusion by suppressing chemokines required for cDC1 recruitment, thereby impairing CD8^+^ T-cell priming and facilitating immune evasion [29]. Consequently, the exclusion or dysfunction of cDCs in tumors correlates with immune escape and therapeutic resistance, particularly to ICIs [30].

### 2.1. Differentiation and Maturation of Natural DC Subsets

DCs were historically classified by ontogeny, but since the rise of single-cell technologies, they are now classified into various subsets based on their functional properties, surface markers, and developmental stages. An overview of human DC subsets can be found in Li et al., while Heras-Murillo et al. further outline the complexity of DC classification across cancers and their roles in adaptive immunity and immunotherapy [31,32]. The primary subsets include cDCs of myeloid lineage—divided into cDC1 and cDC2-plasmacytoid DCs (pDCs) and progenitor DCs, each playing distinct roles in immunity [25,28]. In humans, cDCs account for less than 1% of immune cells in peripheral blood and are characterized by the expression of CD13, CD33, and CD11c with high expression of MHC-class II molecules in a mature state [25,26]. They can further be divided into two major subsets: cDC1 and cDC2. cDC1s, characterized by the expression of CD141 (BDCA-3), are specialized in cross-presentation following immunogenic cell death (ICD), where tumor-associated antigens (TAAs) on MHC class I molecules are presented to CD8^+^ CTLs. cDC1s also play a central role in “re-licensing” antitumor T-cells through the secretion of IL-12, supporting a Th1-type immune response [33,34]. In contrast, cDC2s, defined by the marker CD1c (BDCA-1), are involved primarily in the presentation of antigens to CD4^+^ T-cells via MHC class II molecules, promoting Th2-type immune responses [35,36]. However, emerging evidence indicates that upon acquiring a distinct activation state, cDC2s can also prime cytotoxic CD8^+^ T cells, contributing directly to effective immune responses against viral infections and cancer [37,38]. The recently identified non-conventional type 3 DC (DC3s) subset shares functional similarities with the CD14^+^ cDC2 subgroup but is distinct in phenotype, function, and ontogeny [39,40]. DC3s can prime T cells, promoting Th17 polarization and tissue-resident memory CD8^+^ T cells, yet their role in cancer appears highly context-dependent and may play an immunosuppressive role in some (cold) cancer types. Within the tumor microenvironment—where immune cell composition critically shapes prognosis and response to therapy—DC3s have been associated with favorable outcomes in pancreatic ductal adenocarcinoma and oropharyngeal cancer, but with immunoregulatory or tolerogenic features in melanoma and non-small-cell lung cancer. This evidence could potentially represent a barrier to effective intratumoral cDC therapy. However, the interaction between endogenous DC3s and therapeutically administered cDCs is insufficiently characterized and warrants further investigation [39,40].

Following the described DC differentiation process, the activation of DCs, known as “maturation,” occurs. During maturation, an immature (yet fully developed) DC transitions from its antigen-sampling sentinel mode to a mature antigen-presenting, signaling mode. While both immature and mature DCs capture and process antigens, immature DCs (iDCs) are not fully capable of activating T-cells until they are mature [41]. Upon encountering tumor-associated signals, iDCs migrate to lymphoid organs, where they mature, present antigens with MHC molecules, and upregulate co-stimulatory molecules (CD80, CD86) and cytokines like IL-12, which are essential for T-cell activation [42]. The maturation state of DCs can also influence therapeutic outcomes. iDCs are gaining attention in cancer immunotherapy due to their high phagocytic ability, enabling efficient tumor antigen uptake within the TME. This makes them ideal for in situ vaccination, where iDCs are injected directly into tumors and mature in response to ICD [25,43]. Using iDCs reduces the need for extensive ex vivo manipulation, lowering manufacturing and regulatory complexity and costs. Unlike fully mature DCs, which have a fixed activation profile, iDCs retain plasticity and can be shaped by the tumor microenvironment to elicit a more tailored immune response [25].

In contrast, mature DCs offer advantages related to their potent immunostimulatory capacity and phenotypic stability. They express high levels of MHC class I and II molecules, co-stimulatory receptors, and pro-inflammatory cytokines such as IL-12p70, enabling efficient priming of tumor-specific CD4^+^ and CD8^+^ T-cells while limiting tolerance induction [42,43]. Mature DCs also display enhanced resistance to tumor-mediated immunosuppression and upregulate CCR7, facilitating migration to tumor-draining lymph nodes and supporting systemic anti-tumor immunity [44,45]. However, their reduced antigen uptake capacity, limited adaptability once fully differentiated, and reliance on tightly controlled ex vivo maturation protocols may constrain their effectiveness in certain settings, particularly within heterogeneous or poorly inflamed tumors [46,47].

Collectively, these observations underscore that the strict binary view of “immature equals tolerogenic” and “mature equals immunogenic” is overly simplistic. Instead, a spectrum of DC activation states exists, and therapeutic success may depend more on the timing, location, and microenvironmental context of maturation rather than simply employing fully mature DCs. This dynamic perspective highlights the potential of strategies that harness the plasticity of DCs to generate context-specific immune responses, offering opportunities for more effective and personalized cancer immunotherapies [32].

### 2.2. Importance of cDCs for ICI Efficacy

DCs play a crucial role in the anticancer activity of ICI, including therapeutic mAbs that block PD-1 ligation to PD-L1/-L2, because these therapeutics rely on the presence of tumor-specific T-cell responses that are orchestrated by cDCs [32]. Preclinical studies demonstrate that DCs, particularly cDC1s, are essential for priming and sustaining antitumor T-cell responses, which are critical for ICI efficacy [48,49,50,51]. Tumors in mice lacking CD11c-expressing cells or cDC1s show resistance to ICIs, and cDC1 depletion before or during treatment impairs cancer control [30]. Moreover, key DC-derived factors such as IL-12, CXCL9, and CD5^+^ cDC expression, with the latter specifically in melanoma, enhance the anticancer activity of T-cells and are critical for ICI success in preclinical models [52,53,54].

Additionally, ICI can influence the antitumor function of DCs through multiple direct or indirect mechanisms. Firstly, PD-L1 expression on DCs plays a crucial role in regulating antitumor immunity by restraining T-cell responses during the cross-presentation of tumor antigens. This makes PD-L1 on DCs a key target for anti-PD-1 ICI, as targeting this interaction can significantly enhance T-cell activation [55]. Secondly, PD-L1 blocking antibodies not only inhibit PD-L1-mediated T-cell suppression in the TME and tumor-draining lymph nodes (TDLNs) but also disrupt the PD-L1–CD80 interaction on DCs, promoting CD80 homodimer formation. These homodimers can be removed by Tregs via CTLA4-mediated trogocytosis, reducing CD8^+^ T-cell activation—an effect that can be counteracted by combining anti-PD-L1 with anti-CTLA-4 antibodies to enhance immune responses [55,56,57]. T-cell immunoglobulin and mucin-containing protein-3 (TIM-3) on DCs sequesters damage-associated molecular patterns (DAMPs), particularly extracellular tumor-derived DNA and the alarmin HMGB1, thereby limiting DNA uptake and suppressing activation of the cGAS–STING pathway. Blocking TIM3 enhances DAMP recognition, activating DCs and strengthening the anticancer T-cell response, making TIM3 inhibition a promising strategy to improve ICI efficacy [58,59,60,61]. Lastly, the effectiveness of agonistic anti-CD40 antibodies in enhancing ICIs relies on DC licensing, whereby CD40 engagement drives DC activation and maturation, allowing them to effectively prime T-cells and amplify the antitumor immune response. Combining CD40 agonists with ICIs results in a more powerful and lasting immune attack on tumors [36,62]. Hence, strategies that restore or enhance DC function—through progenitor manipulation or direct DC administration—therefore hold promise for improving cancer immunotherapy outcomes [32].

### 2.3. Exploiting cDCs as Key Orchestrators for Optimized Therapeutic Cancer Vaccination

**Figure 1:**
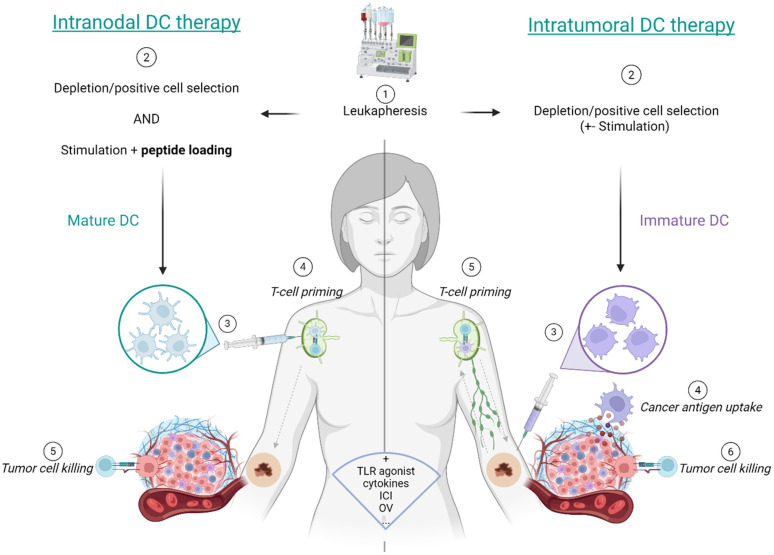
In both approaches, patients undergo leukapheresis followed by monocyte depletion and positive DC selection (e.g., CD14/CD19 depletion, CD1c^+^ enrichment). DCs may be stimulated ex vivo (e.g., with GM-CSF or mRNA/protamine), whereas intratumoral (IT) delivery can use unstimulated, immature DCs. In intranodal (IN) therapy, DCs are peptide-loaded (e.g., gp100) prior to injection. IT injection allows immature DCs to capture tumor antigens in situ, migrate to draining lymph nodes for T-cell priming, and subsequently return to the TME to mediate tumor cell killing; IN delivery bypasses this IT antigen acquisition step, potentially limiting local tumor cytotoxicity. Combinatorial therapies, including immune checkpoint blockade, oncolytic viruses, or Toll-like receptor (TLR) agonists as local adjuvants to promote DC maturation and enhance antigen presentation within the TME, can be added to further strengthen DC activation and antitumor immunity. Abbreviations: DC: dendritic cell, TLR: Toll-like receptor, ICI: immune checkpoint blockade, OV: oncolytic viruses. (Created in BioRender. Vounckx, M. (2026) https://BioRender.com/idtz7y9; accessed on 29 April 2026).

A key consideration in DC therapy is the route of administration, which is elaborated in Figure 1. Intranodal (IN) injection delivers DCs directly into lymph nodes, enhancing DC-T-cell interaction and systemic immune activation. It is clinically favored for its reproducibility and safety. However, IN injection has a limited impact on the TME since it bypasses the tumor site and depends on potentially inefficient DC migration from the lymph node to the tumor, which may fail to reverse local immunosuppression. Moreover, it depends on DCs pre-loaded with selected antigens, therefore limiting the range of immune responses, which is particularly unfavorable in tumors with intrinsic heterogeneity [42,63]. IT delivery of cDCs enables in situ vaccination while simultaneously reprogramming the TME [64]. Importantly, IT therapy promotes a T-cell response directed against the full repertoire of antigens actively expressed by the tumor at the time of treatment, thereby fostering a more tumor-oriented and dynamically adaptive immune response compared with DC vaccines loaded ex vivo with selected antigens. Moreover, IT cDCs can directly activate NK cells, which in turn secrete cytokines such as CCL5, XCL1, and Flt3L that attract endogenous DCs and enhance cross-priming [29]. NK–DC crosstalk also promotes DC maturation and Th1 polarization, amplifying adaptive immune responses within the tumor itself [65]. Additionally, allogeneic IT cDCs can trigger immune activation without specific antigen targeting. They promote chemokine-driven T-cell infiltration and may synergize with other therapies to enhance local and systemic immunity [42,64].

During the past two decades, several early-phase and one phase III clinical studies have explored the therapeutic potential of DC-based IT immunotherapies in melanoma and other solid tumors. For example, in 2007, the mDC-PROS1 and mDC-PROS2 trials in metastatic prostate cancer evaluated CMRF-56-selected CD1c^+^ cDC vaccines, administered either intradermally or intravenously. These studies demonstrated feasible cell yields but limited purity and predominantly semi-mature DC phenotypes, highlighting early manufacturing and activation challenges in this setting [66]. Table 1 gives an overview of all clinical trials in melanoma involving IT or IN human blood cDCs. At Radboud University Medical Center, a series of trials has progressively refined the use of antigen-loaded mature DCs. A phase I trial by Schreibelt et al. employing CD1c-selected and mRNA- or peptide-pulsed DCs enrolled 14 patients with stage IV melanoma [67]. Autologous CD1c^+^ myeloid DCs (3–10 × 10^6^ cells) loaded with gp100 and tyrosinase were injected following minimal ex vivo culture; the regimen was well tolerated and induced multifunctional CD8^+^ T-cell responses in three of the long-term progression-free survivors (12–35 months). Although objective tumor regressions were limited, these results established the feasibility and evidence of immune activation [67]. Building on this foundation, Bol et al. reported the first phase III trial using a mature, protamine/mRNA-stimulated DC vaccine targeting multiple melanoma antigens (gp100, tyrosinase, NY-ESO-1, and MAGE family) [68]. This MIND-DC randomized phase III trial (*n* = 148, stage IIIB/C melanoma) compared IN administration of autologous CD1c^+^/pDCs with placebo. Further, 67.1% of treated patients developed functional antigen-specific T-cell responses, with a 2-year recurrence-free survival of 36.8% versus 46.9% in the control group (HR 1.25; *p* = 0.31), with a median recurrence-free survival (RFS) of 12.7 versus 19.9 months. Grade 3–4 adverse events occurred in only 5% of treated patients, confirming a favorable safety profile. Despite clear immunologic activation, this study did not achieve its primary clinical endpoint, highlighting the need for optimized combination strategies and biomarker-driven patient selection [68]. The parallel combiDC-MEL1 trial (NCT02574377), designed to test mixed pDC/cDC formulations, was discontinued prematurely but provided important translational insights, revealing unequal contributions of DC subsets to antitumor immunity and informing the rational design of future DC vaccine strategies [69].

### 2.4. Exploiting IT cDCs to Reinvigorate the Cancer Immune Cycle

Overcoming immune suppressive mechanisms that counteract responses to ICI is a central goal of evolving novel immunotherapy approaches. IT immunotherapy, by directly targeting the tumor site, aims to reinvigorate the cancer-immunity cycle where it is locally disrupted. Unlike systemic approaches, IT delivery minimizes the potential for off-tumor toxicities, allows lower dosing (while achieving high local concentrations), and exploits the potential for localized activation of both innate and adaptive immunity [73]. Most desirable IT strategies may induce abscopal effects, leading to a systemic antitumor immune response covering distant metastatic sites [73,74,75,76,77,78]. Thus, IT administration of DCs or interventions that attract cDCs and provide adequate maturation and activation signals can represent a rational and potentially transformative approach for combinatorial anti-cancer immunotherapy.

**Figure 2:**
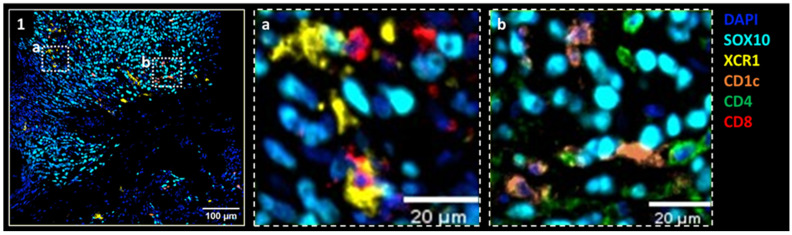
Representative Opal multiplex IHC images of melanoma tissue illustrate the spatial distribution of conventional dendritic cell (cDC) subsets within the tumor microenvironment (TME) following intratumoral (IT) therapy with autologous CD1c (BDCA-1)^+^/CD141 (BDCA-3)^+^ cDCs. Image 1 shows the co-existence of XCR1^+^ cDC1 (yellow) and CD1c^+^ DCs (orange) within the SOX10^+^ tumor area (light blue). Images a and b provide higher-magnification views, highlighting CD1c^+^ cDC2 in proximity to CD4^+^ T cells (green) and XCR1^+^ cDC1 in close contact with CD8^+^ T lymphocytes (red), respectively, within the SOX10+ tumor area. These images are reported based on unpublished data subsequent to a presented congress abstract [83].

Accumulating evidence suggests that naturally circulating cDCs, particularly cDC1 (CD141^+^) and cDC2 (CD1c^+^) subsets, may provide superior antigen presentation and T-cell priming capacities compared to moDCs. Their clinical relevance is further supported by The Cancer Genome Atlas, which links cDC—rather than moDC—infiltration to improved survival in various tumor types [28,79,80]. Naturally circulating autologous cDCs have shown promise beyond melanoma; in a phase 1 trial in metastatic castration-resistant prostate cancer, DCs combined with cryoimmunotherapy were safe with a median OS of 40.7 months, and recurrent glioblastoma, where intracranial cDCs with nivolumab and ipilimumab were feasible and associated with encouraging survival based on unpublished data as reported in a congress abstract [81,82]. Furthermore, direct cDC isolation is now feasible by immunomagnetic bead isolation on the CliniMacs^®^ platform (Miltenyi Biotech) and allows better standardization for clinical trials and future routine use [66]. In parallel, advances in spatial proteomics technologies such as Opal multiplex immunohistochemistry (mIHC) allow the detection of multiple biomarkers on the same tissue section. This approach offers a major advantage for visualizing and tracking cDC subsets and immune response within the TME during IT treatment, as illustrated in Figure 2. Such spatially resolved analysis facilitates a more refined characterization of the TME and offers mechanistic insight into how cDCs may orchestrate local immune activation and antitumor responses.

A key strategy to enhance the efficacy of IT cDC therapy is its combination with other modalities such as systemic ICI, radiation- or cryotherapy, chemotherapy, or additional IT therapies including oncolytic viruses (OVs) or synthetic adjuvants. IT immunotherapy has shown potential synergy when combined with ICI, as investigated in the Gustave Roussy HIT-IT trial in solid tumors [84]. Talimogene laherparepvec (T-VEC) is the first IT oncolytic viral therapy to gain approval for use in melanoma, which showed early promise but failed to improve progression-free survival (PFS) or overall survival (OS) as a first-line therapy for patients diagnosed with unresectable advanced melanoma when combined with pembrolizumab in the phase III MASTERKEY 265 study [85]. Importantly, this trial combined IT T-VEC with systemic ICI, a design that differs from IT cDC–oncolytic virus strategies discussed later in the review and should not be interpreted as a broad limitation of oncolytic virus-based IT approaches.

A recent series (Table 1) of phase I studies has explored IT cDC therapy in combination with ICI. Schwarze et al. demonstrated that IT injection of autologous immature CD1c^+^ DCs combined with an OV (T-VEC) or ICI (anti-CTLA4/PD-1) was feasible and well tolerated [70,71,86]. A cohort of 13 advanced, heavily pretreated melanoma patients received IT injections of CD1c^+^ and CD141^+^ cDCs following T-VEC preconditioning. Cohorts escalated DC dosing up to 10 × 10^6^ cells; median T-VEC injections were six (range 3–8). Two patients achieved a durable complete response (ongoing at 33 and 35 months), one patient achieved an unconfirmed partial response, and others achieved mixed responses or stable disease. Fatigue (85%), injection-site pain/redness (69%), fever (62%), and chills (46%) were the most common adverse events. These trials showed biological evidence of immune activation within injected lesions, accompanied by occasional partial and durable systemic responses in PD-1-refractory melanoma [70,71,87]. Building on these findings, Tijtgat et al. reported the first-in-human trial of IT CD1c^+^/CD141^+^ cDCs combined with IT ipilimumab and AS01^b^ adjuvant injections plus low-dose intravenous nivolumab (*n* = 8) in ICI- and BRAF/MEK-inhibitor refractory patients [72]. AS01^b^ is a synthetic liposome-based immunostimulatory adjuvant containing monophosphoryl lipid A (MPL) and QS-21 that enhances innate immune activation and antigen presentation. A disease control rate (DCR) of 50% was achieved with several durable complete or partial responses with a median PFS of 24.1 weeks and median OS of 41.9 weeks; no unexpected safety signals occurred. Responding lesions showed marked immune activation characterized by increased CD8^+^ T-cell infiltration and type I IFN signatures, supporting the potential of this approach to overcome local immune suppression and restore antitumor reactivity in ICI-refractory disease [72]. In a second cohort, patients received intensified weekly administration of AS01^b^, which recapitulated the initial results, with several patients demonstrating robust clinical responses based on preliminary unpublished data as reported in a congress abstract [83]. Based on this promising activity, a phase II trial, now fully recruited, is being analyzed to validate and expand these findings.

Beyond T-VEC, next-generation OVs such as RP1 (vusolimogene oderparepvec), engineered to express GM-CSF and the fusogenic protein GALV-GP-R, have demonstrated enhanced immunogenicity and promising activity in PD-1 refractory melanoma [88]. In the IGNYTE phase I/II trial (NCT03767348), RP1 plus nivolumab achieved a 36% objective response rate (ORR), including CR in patients with visceral metastases and systemic effects in non-injected lesions [89]. Therefore, further investigation of the combination of such OV with IT administration or attraction of cDC warrants consideration. Hence, combining defined cDC subsets with OVs and/or IT adjuvants, and PD-1 blockade—as in the UZ Brussel and Radboud programs—highlights the shift of DC-based IT therapy toward a rational combinatorial strategy to reprogram the TME and overcome ICI resistance.

## 3. Conclusions

IT DC therapy represents an innovative and promising strategy to reinvigorate antitumor immunity in melanoma, and to a lesser extent in other solid tumors. Compared with traditional moDC vaccines, blood-derived cDCs display superior immunogenicity, cross-priming capacity, and clinical relevance. IT delivery not only facilitates in situ antigen capture and maturation but also remodels the immunosuppressive TME and enhances systemic immune activation. The use of immature DCs injected directly into tumors, without prior antigen loading, supports a potent in situ vaccination effect, particularly when integrated with preconditioning regimens such as ICI. Autologous blood-derived cDCs currently represent the most clinically advanced and biologically compatible platform, but broader implementation will require harmonized manufacturing protocols and standardized potency assays. Finally, preclinical and early clinical data suggest that ICI may act as an effective preconditioning or concurrent partner to IT cDC by relieving T-cell exhaustion and enhancing DC-mediated priming, but the optimal sequencing strategy—preconditioning, concurrent, or sequential—has not yet been defined. Future clinical trials should focus on optimizing the combination and sequencing of IT cDC therapy with other immunomodulatory treatments, such as ICI and/or OV or adjuvants. Together, these strategies may help establish IT DC therapy as a central approach for cancer immunotherapy.

## Figures and Tables

**Table 1:** Overview of all clinical trials of intratumoral (IT) and intranodal (IN) naturally circulating DC in melanoma.

Phase (Ref.)	NCT Number	Melanoma Stage	Institution	DC Maturation	Maturation Stimulus	DC Type	Antigens	Immune Modulators	Route
III ^1^	NCT00243529	IIIB–C	Radboud UMC, Nijmegen, The Netherlands	Matured	Protamine/ mRNA	Combi: CD19^−^, CD14^−^ depletion, CD1c^+^	gp100, tyrosinase, NY-ESO-1, MAGE-C2, MAGE-A3		IN
I & II ^2^ (prematurely stopped)	NCT00940004	III	Radboud UMC, Nijmegen, The Netherlands	Matured	–	CD1c^+^ mDC, pDC, or combination	–		
I ^3^	NCT01530698	IV	Radboud UMC, Nijmegen, The Netherlands	Matured	GM-CSF	CD19^−^ depletion, CD1c^+^	gp100, tyrosinase		
I ^4^	NCT02070406	IV	UZ Brussel, Belgium	Immature	None	CD19^−^, CD14^−^ depletion, CD1c^+^ + BDCA-3^+^ (CD141^+^)		T-VEC	IT
I ^5^	NCT02649829	IV + solid tumors	UZ Brussel, Belgium	Immature	None	CD19^−^, CD14^−^ depletion, CD1c^+^ + BDCA-3^+^ (CD141^+^)		Anti-CTLA-4/PD-1/PD-L1	
I ^6^	NCT03747744	III–IV	UZ Brussel, Belgium	Immature	None	CD19^−^, CD14^−^ depletion, CD1c^+^ + BDCA-3^+^ (CD141^+^)		Adjuvant AS01^b^	

^1^ *K. F. Bol, Nat Commun 2024 [68],* ^2^ *combiDC-MEL1 (NCT-02574377) [69],* ^3^ *G. Schreibelt, Clin Cancer Res 2016 [67],* ^4^ *J. K. Schwarze, J Immunother Cancer 2022 [70],* ^5^ *J. K. Schwarze, Vaccines (Basel) 2020 [71],* ^6^ *J. Tijtgat, J Immunother Cancer 2024 [72].*

## Data Availability

This review is based on previously published data and on unpublished data presented in congress abstract, as clearly indicated in the text of the manuscript. All data sources are cited within the article, and no new data were generated.

## Abbreviations

The following abbreviations are used in this manuscript

ICI	Immune Checkpoint Inhibition
PD-(L)1	Programmed Cell Death Protein 1/Programmed Death-Ligand 1
CTLA-4	Cytotoxic T-Lymphocyte-Associated Protein 4
LAG-3	Lymphocyte Activation Gene 3
TIM3	T-cell immunoglobulin and mucin containing protein-3
mAb	Monoclonal Antibodies
TMB	Tumor Mutation Burden
NSCLC	Non-Small Cell Lung Carcinoma
cDC	Conventional Dendritic Cells
MHC	Major Histocompatibility Complex
TME	Tumor Microenvironment
APC	Antigen-Presenting Cells
DC	Dendritic Cell
moDC	Monocyte-Derived Dendritic Cells
TAA	Tumor-Associated Antigens
GM-CSF	Granulocyte-Macrophage Colony-Stimulating Factor
IL-4	Interleukin-4
PD-1	Programmed Cell Death Protein 1
ICOSL	Inducible T-cell Co-Stimulator Ligand
CTL	Cytotoxic T Lymphocyte
cDC1	Conventional Dendritic Cell Type 1
cDC2	Conventional Dendritic Cell Type 2
TGF-β	Transforming Growth Factor Beta
VEGF	Vascular Endothelial Growth Factor
Treg	Regulatory T-Cell
MDSC	Myeloid-Derived Suppressor Cells
pDC	Plasmacytoid Dendritic Cells
ICD	Immunogenic Cell Death
iDC	Immature Dendritic Cell
DAMP	Damage-Associated Molecular Patterns
HMGB1	High Mobility Group Box 1
IN	Intranodal
IT	Intratumoral
TLR	Toll-Like Receptor
OV	Oncolytic Virus
RFS	Recurrence-Free Survival
T-VEC	Talimogene Laherparepvec
OS	Overall Survival
PFS	Progression-Free Survival
MPL	monophosphoryl lipid A
DCR	Disease Control Rate
CR	Complete Response
ORR	Objective Response Rate
RP1	Vusolimogene Oderparepvec
